# Resisting the urge to smoke: inhibitory control training in cigarette smokers

**DOI:** 10.1098/rsos.170045

**Published:** 2017-08-16

**Authors:** Sally Adams, Claire Mokrysz, Angela S. Attwood, Marcus R. Munafò

**Affiliations:** 1 UK Centre for Tobacco and Alcohol Studies and Department of Psychology, University of Bath, Bath, UK; 2 Clinical Psychopharmacology Unit, University College London, London, UK; 3 MRC Integrative Epidemiology Unit (IEU), University of Bristol, Bristol, UK; 4 UK Centre for Tobacco and Alcohol Studies and School of Experimental Psychology, University of Bristol, Bristol, UK

**Keywords:** cognitive training, nicotine, cigarette smokers

## Abstract

Impaired response inhibition is an important factor in tobacco dependence. We examined the effects of inhibitory control training (ICT) on inhibition, smoking resistance and cigarette use. Smokers (*n* = 55) abstained from smoking for 12 h prior to testing. On the test day, participants recorded cigarette use and completed pre-training measures of global and cue-specific (smoking-related) response inhibition. Participants were randomized to either an active or a control ICT group. The active group was required to repeatedly inhibit a response towards smoking cues (100%), while the control group was required to inhibit a response towards smoking and neutral cues with equal frequency (50%). Participants performed post-training measures of response inhibition, smoking resistance and cigarette use. Inhibition data did not indicate time (pre-training, post-training) × group (active training, control training) or time × group × cue (smoking, neutral) interactions. There was weak evidence that smokers in the active group were more likely to resist smoking than those in the control group. Cigarette use data did not indicate a time × group interaction. Our data suggest that ICT may enhance the ability to resist smoking, indicating that training may be a promising adjunct to smoking pharmacotherapy.

## 1. Background

Impaired response inhibition is an important etiological factor in models of tobacco dependence [1]. Smokers may have difficulty in resisting smoking urges, especially when confronted with smoking-related cues. However, no smoking cessation programmes have focused on improving inhibitory control towards smoking-related stimuli in cigarette smokers.

Smokers have greater difficulty in inhibiting responses than non-smokers [2], and more dependent smokers show lower inhibition capacity than lighter, less nicotine-dependent smokers [1,3]. These findings suggest that individual differences in inhibitory control may predict cigarette use and level of nicotine dependence. Response inhibition in smokers is further attenuated during smoking cessation [4–6], while nicotine replacement therapy in abstinent smokers increases inhibitory control compared with placebo [7,8]. These studies suggest that abstinence from nicotine underlies reduced response inhibition during smoking cessation, rather than non-nicotine related factors (e.g. habit) [9]. Impaired inhibitory control during abstinence may therefore be a potential mechanism underlying relapse.

Recent research has begun to explore the possibility of using inhibitory control training (ICT) to reduce alcohol and food intake. Several studies, together with two recent meta-analyses [10,11], indicate that a computer test that requires participants to repeatedly inhibit a response towards relevant stimuli (e.g. alcohol-related cues) results in behaviour change (e.g. reduced alcohol consumption). However, it is not clear how ICT modifies behaviour. Research has supported three key mechanisms in the modification of behaviour: (i) devaluation of relevant stimuli (e.g. alcohol-related cues), (ii) development of a stop-stimulus response, and (iii) strengthening of an inhibitory response. ICT has been shown to lead to stimulus devaluation [12–14], but evidence has suggested that this effect may be influenced by how devaluation is assessed. While a recent meta-analysis [11] of studies primarily using implicit association measures of stimulus devaluation did not show clear evidence of an overall effect of ICT on stimulus devaluation, other studies using explicit measures of devaluation have demonstrated that ICT leads to changes in evaluation [15] and value [16] of stimuli. Other studies have shown that ICT may exert its effects by strengthening inhibitory responding for stimuli consistently paired with a stopping response [17]. Verbruggen & Logan [17] suggest that by repeatedly inhibiting a response towards a relevant stimulus, the stimulus may become associated with an inhibitory stop ‘tag’, leading to subsequent devaluation. However, it is not clear whether this stop-stimulus response is specific to the stimulus or may reflect a more global strengthening of inhibitory response. A recent theoretical model suggests that ICT leads to devaluation of relevant stimuli only [18].

To date, no studies have explored the possible mechanism underling ICT in cigarette smokers. Therefore, in this study, we used a non-cued test of inhibitory control and a test of inhibitory control towards smoking versus neutral stimuli to explore whether ICT improves inhibitory control globally or towards condition-specific stimuli. To our knowledge, this study is the first to examine the effectiveness of ICT, and the mechanism underlying training, in cigarette smokers. Better understanding of the mechanism underlying ICT will provide important insights into how inhibitory control influences smoking behaviour, and may enable the development of novel interventions. We hypothesized that ICT would improve response inhibition towards smoking-related cues in smokers, and that ICT would improve the ability to resist smoking and reduce cigarette use.

## 2. Material and methods

### 2.1. Design

A double-blind between-subjects design, with one between-subjects factor of training (active, control) and a within-subjects factor of time (pre-training, post-training).

### 2.2. Participants

Nicotine-dependent cigarette smokers were recruited from students and staff at the University of Bristol and members of the general public. Smokers were required to smoke a minimum of 10 cigarettes (or 15 roll-ups) per day, and smoke within 1 h of waking. Participants received £20 each for participation.

### 2.3. Materials

#### 2.3.1. Questionnaires

Questionnaire measures included: Fagerström Test of Nicotine Dependence (FTND) [19], Readiness to Quit ladder (RQL) [20], Questionnaire of Smoking Urges-Brief (QSU-Brief) [21], Barratt Impulsivity Scale (BIS-II) [22], Zuckerman–Kuhlman Personality Questionnaire Impulsive Sensation Seeking subscale (ImpSS) [23], Minnesota Nicotine Withdrawal Scale (MNWS) [24] and Visual Analogue Scales (VAS) mood, anxiety and craving.

#### 2.3.2. Cigarette use

Cigarette use was measured using a timeline follow-back (TLFB) procedure, administered via questionnaire [25]. This asks participants to self-report cigarette use, using prompts (e.g. think about occasions on which you would have smoked, did you see friends, etc.).

#### 2.3.3. Go/No-Go test/training stimuli

No-Go stimuli (presented on 50% of Go/No-Go (GNG) blocks) consisted of black arrows (5.2 × 3 cm) presented in the centre of a white screen. Go stimuli (presented on 50% of GNG blocks and 100% of Go-only blocks) were vertical-presented arrows and No-Go stimuli were the same arrows rotated at 45° clockwise. Cue-specific GNG stimuli were a circle and a square (e.g. Go = circle, No-Go = square, with instructions counterbalanced) presented in one of the four corners of the cue-specific images. Cue-specific images (25.6 × 22.1 cm) were four cigarette-related images (e.g. people holding/smoking cigarettes) and four neutral images (e.g. people holding/using stationery) adapted from Gilbert [26] presented in the centre of a black screen.

### 2.4. Procedure

All participants were tested between 09.00 and 12.00 h in a laboratory in the School of Experimental Psychology at the University of Bristol. Participants provided informed consent and completed a screening process to confirm good physical and psychiatric health. Prior to the test session all participants were asked to abstain from smoking for 12 h (overnight abstinence, verified by exhaled CO test, with a cut-off of less than 10 ppm for study inclusion). Baseline questionnaire measures were completed (FTND, RQL, BIS-II, ImpSS, QSU-Brief, MNWS and VAS). Participants were asked to self-report cigarette use for the past 7 days using the TLFB, followed by pre-training GNG tests. GNG tests were included to measure global and cue-specific inhibitory control before and after training.

The global GNG test was adapted from [27]. In the test, participants were instructed to respond to trials in which the arrow was presented vertically (Go trials) by pressing the spacebar on the keyboard, and to not respond to trials in which the arrow was presented at a rotated angle (No-Go trials). Each trial began with a central fixation cross presented for 500 ms, followed by an arrow stimulus presented for 2000 ms, with an inter-stimulus interval of 1500 ms. The test comprised five blocks, presented in a sequential order as follows: Block 1 = Go and No-Go (GNG), Block 2 = G, Block 3 = GNG, Block 4 = G and Block 5 = GNG. Each block consisted of 24 trials, with GNG blocks including 12 Go trials and 12 No-Go trials. Test completion was self-paced, with a brief pause between blocks. Inclusion of Block 1 served as a baseline for performance, with Go-only blocks included to encourage habitual response to Go stimuli. The duration of the test was approximately 5 min.

In the cue-specific GNG test, participants were instructed to press the space bar when a Go cue was displayed, and to withhold a response (i.e. press nothing) when a No-Go cue was presented. The GNG cues were a circle or a square (with the Go versus No-Go counterbalanced across participants) presented in one of the four corners of a smoking-related or neutral stimulus image. In the test, smoking-related and neutral cues were paired with the Go and No-Go cue with equal frequency. No-Go cues were infrequent (25% of trials). During each trial a stimulus was presented with a cue for 1500 ms. A green circle was displayed for 500 ms after a correct response and a red cross displayed for 500 ms after an incorrect response. The test consisted of two blocks of 64 trials. Test completion was self-paced, with a brief pause between blocks. Trials were presented in a random order and the duration of the test was approximately 5 min. Following completion of test GNG tasks participants were randomized to complete active or control GNG training.

In the active GNG training, smoking-related cues were paired with the No-Go cue for all trials (100%). In the control GNG training, smoking-related and neutral cues were paired with the No-Go cue with equal frequency. The procedure of each trial was the same as that described for the cue-specific GNG test, except the training consisted of four blocks of 64 trials. Training completion was self-paced, with a brief pause between blocks. The duration of the training was approximately 30 min. Following training, participants completed post-training GNG tests and the smoking resistance test.

In the smoking resistance test, participants were instructed that they ‘could accumulate a monetary reward by not smoking or earn a cigarette by pressing a button’. Participants accumulated money (£0.10) every 30 s (up to a maximum duration of 20 min), until they first pressed the button to receive a cigarette. The accumulative monetary amount was displayed on a computer screen until the spacebar key was pressed for the first time. Once the spacebar was pressed, participants could not earn any more monetary rewards, but could receive a cigarette reward. We used time to first button press as a marker of smoking resistance. Participants had the opportunity to earn a maximum of £4. However, regardless of when a participant pressed to receive a cigarette reward, all participants received the maximum amount on completion of the study. Following completion of this task participants completed questionnaire measures (QSU-Brief, MNWS, VAS) and were given the TLFB questionnaire to record cigarette use for the next 7 days. On return of the questionnaire, participants were reimbursed for their time and received a full debrief.

### 2.5. Data analysis

GNG commission errors were analysed within a 2 × 2 design, with training group (active, control) as a between-subjects factor and time (pre-training, post-training) as a within-subjects factor and a within-subjects factor of cue type (smoking, neutral) for the cue-specific GNG. Commission error data were square root transformed due to positive-skew. Cigarette use data were examined by ANOVA of cigarettes per week, with a between-subjects factor of training group. Cigarette use data for two participants were missing.

Smoking resistance data (i.e. latency to ‘first button press’) were bimodally distributed and were therefore converted to a binary variable, with responses categorized as ‘button pressed’ (smoked) or ‘button not pressed’ (abstained). Data were analysed using logistic regression, with smoking resistance coded as smoked or abstained, and training group as a predictor. A *post hoc* sensitivity analysis, for our primary outcome of ICT on cigarette use, indicated that the study had 80% statistical power at an alpha level of 5% to detect an effect size of *f* = 0. 33 for the training group by cigarette use interaction effect. Analyses were conducted using IBM SPSS Statistics v.22.

## 3. Results

### 3.1. Characteristics of participants

Participants (*n* = 55, 29 male) were, on average, aged 24 years (s.d*.* = 8, range 18–50), had a FTND score of 4 (s.d. = 1, range 2–7) and smoked on average 13 cigarettes per day (s.d. = 4, range 7–21). Table 1 shows characteristics of participants by training group.

**Table 1.** Characteristics of participants by training group. Values are expressed as mean (s.d.).

	active training (*n* = 27)	control training (*n* = 28)
age (years)	23 ± 7	25 ± 8
cigarettes (per day)	14 ± 4	14 ± 4
FTND	5 ± 2	4 ± 1
QSU	46 ± 2	44 ± 11
MNWS	19 ± 9	17 ± 9
BIS-II	70 ± 11	111 ± 188
IMPSS	12 ± 4	13 ± 4
VAS-happy	57 ± 15	55 ± 15
VAS-dowsy	53 ± 24	40 ± 26
VAS-depressed	27 ± 21	24 ± 19
VAS-anxious	32 ± 24	30 ± 23
VAS-energetic	43 ± 19	42 ± 22
VAS-irritable	41 ± 22	34 ± 22
VAS-craving a cigarette	74 ± 11	69 ± 18

### 3.2. Cigarette craving and withdrawal

ANOVA of mean QSU-Brief scores indicated a main effect of time (*F*_1,53_ = 26.98, *p* *<* 0.001, *η*_2_ = 0.34), such that craving increased from pre- to post-training. ANOVA of mean MNWS scores indicated a main effect of time (*F*_1,53_ = 18.58, *p* < 0.001, *η*_2_ = 0.26) that was qualified by an interaction between time × training group (*F*_1,53_ = 5.79, *p* = 0.020, *η*_2_ = 0.10). *Post hoc* tests indicated a main effect of time for participants in the control condition such that withdrawal increased from pre- to post-training (*F*_1,27_ = 22.64, *p* < 0.001, *η*_2_ = 0.46), but not for participants in the active condition (*F*_1,26_ = 1.81, *p* = 0.19, *η*_2_ = 0.07).

### 3.3. Mood

ANOVAs of mean VAS scores indicated main effects of time on anxiety, cigarette craving, drowsiness, happiness and irritability (*p* < 0.05), where anxiety, cigarette craving and drowsiness increased from pre- to post-training and happiness decreased. The main effect of time on drowsiness was qualified by an interaction between time × training group (*F*_1,53_ = 5.57, *p* = 0.022, *η*_2_ = 0.10). *Post hoc* tests revealed a main effect of time for participants in the control condition only, such that drowsiness increased from pre- to post-training (*F*_1,27_ = 10.39, *p* = 0.003, *η*_2_ = 0.28), but not for participants in the active condition (*F*_1,26_ = 0.07, *p* = 0.80, *η*_2_ = 0.003)

### 3.4. Cue-specific Go/No-Go

ANOVA of commission errors indicated a main effect of time (*F*_1, 53_ = 11.00, *p* *=* 0.002, *η*_2_ = 0.17), such that all participants made more commission errors at post-training compared with pre-training. There were no further main effects or interactions (*p* > 0.12).

### 3.5. Global Go/No-Go

ANOVA of commission errors indicated a main effect of time (*F*_1,53_ = 4.73, *p* *=* 0.034, *η*_2_ = 0.08), such that all participants made more commission errors at post-training compared with pre-training. There were no further main effects or interactions (*p* > 0.59).

### 3.6. Cigarette use

ANOVA indicated that the number of cigarettes smoked post-training did not differ according to training group, after adjusting for cigarettes smoked pre-training (*F*_1,50_ = 0.002, *p* *=* 0.97, *η*_2_ < 0.001).

### 3.7. Smoking resistance

Logistic regression of smoking resistance data indicated weak evidence that active training was associated with greater odds of abstinence (OR = 2.65, 95% CI 0.844–8.336, *p* = 0.095). Adjustment for baseline impulsivity, cigarette craving, nicotine withdrawal and nicotine dependency did not substantially alter the point estimate, but resulted in weaker statistical evidence (OR = 2.33, 95% CI 0.718–7.545, *p* = 0.159). Table 2 shows smoking resistance by training group.

**Table 2.** Smoking resistance by training group. Number of participants who smoked or abstained on the smoking resistance task by training group (ICT, control).

	training group	
	ICT	control
abstained	*n* = 13	*n* = 7
smoked	*n* = 14	*n* = 20

## 4. Discussion

Our results do not suggest that ICT improves GNG response inhibition towards smoking-related cues in nicotine-dependent cigarette smokers. We also did not observe an effect of ICT on reduction of cigarette use. However, we did observe some weak evidence that ICT may improve smoking resistance, suggesting that it may warrant further investigation, particularly in treatment-seeking smokers.

In contrast with previous studies of alcohol and food use [12,28], we did not observe that ICT reduced cigarette use. However, the majority of previous ICT studies have been conducted in young, healthy female students [11], with few criteria for inclusion/exclusion. Our participants were nicotine-dependent smokers, who were unmotivated to quit. Evidence has indicated that smoking cessation treatment response is highly influenced by readiness to quit smoking [29], which may have prevented a reduction in cigarette use being observed in our study. In line with one previous study [13], we did not observe evidence for ICT operating via a mechanism of improving inhibitory control over response towards relevant stimuli. This finding is in contrast to the proposed effect of a GNG manipulation on strengthening inhibition behaviour towards stimuli that are consistently paired with the no-go stopping response [17].

We observed weak evidence for ICT improving smoking resistance. This small effect may in part be explained by the design of our control training. Our control training task had a 50% inhibition contingency and included smoking-related stimuli, requiring non-active training participants to engage in inhibition towards some smoking stimuli. This conservative design may have prevented a stronger effect from being observed. Additionally, a lack of training effect on response inhibition towards smoking-related cues may reflect the 75% response/25% inhibition contingency on our test measure of response inhibition, which may have increased pre-potent response and obscured training effects. Further research is required to examine the impact of different methodological factors on ICT effectiveness.

Our findings should be considered in light of the following limitations. First, all participants were exposed to the 50% training contingency (e.g. equal inhibition of smoking and neutral cues) in the post-training test of cue-specific inhibitory control. This may have compromised the effects of the active training. However, the training was comprehensive with 256 trials and a duration of 30–40 min. Second, our sample was limited to non-treatment seeking nicotine-dependent cigarette smokers. It is unknown how non-dependent or nicotine-dependent, treatment-seeking smokers would respond to ICT. Third, our *post hoc* sensitivity analysis revealed that the present study may have been underpowered to detect an effect of ICT on cigarette use. However, smaller effect sizes, which our study lacked the power to detect, may still be potentially clinically meaningful.

## 5. Conclusion

We did not observe clear evidence that ICT strengthens inhibitory response to relevant stimuli following GNG training. However, our results provide some evidence that ICT may enhance the ability to resist smoking. Although this evidence is weak, this effect was observed after only one session of ICT. Further research is required to determine the mechanism underlying ICT in cigarette smokers and to clarify the effectiveness of administering extended ICT to smokers motivated to stop smoking.

## Data accessibility

The data that form the basis of the results presented here are available on request from the data.bris University of Bristol Research Data Repository (http://dx.doi.org/10.5523/bris.y7288gnrb7g51m2u3koi7tbyz).
